# Accelerated apoptotic death and *in vivo* turnover of erythrocytes in mice lacking functional mitogen- and stress-activated kinase MSK1/2

**DOI:** 10.1038/srep17316

**Published:** 2015-11-27

**Authors:** Elisabeth Lang, Rosi Bissinger, Abul Fajol, Madhuri S. Salker, Yogesh Singh, Christine Zelenak, Mehrdad Ghashghaeinia, Shuchen Gu, Kashif Jilani, Adrian Lupescu, Kathleen M. S. E. Reyskens, Teresa F. Ackermann, Michael Föller, Erwin Schleicher, William P. Sheffield, J. Simon C. Arthur, Florian Lang, Syed M. Qadri

**Affiliations:** 1 Department of Physiology, University of Tübingen, Gmelinstr. 5, 72076 Tübingen, Germany; 2 Department of Gastroenterology, Hepatology and Infectious Diseases, University of Düsseldorf, Moorenstrasse 5, 40225 Düsseldorf, Germany; 3 Charité Medical University Berlin, Charitéplatz 1, 10117 Berlin, Germany; 4 Life Sciences Institute, Zhejiang University, Hangzhou, Zhejiang 310058, China; 5 Department of Biochemistry, University of Agriculture, 38040 Faisalabad, Pakistan; 6 MRC Phosphorylation Unit, University of Dundee, Dow Street, Dundee DD1 5EH, United Kingdom; 7 Division of Cell Signaling and Immunology, College of Life Sciences, University of Dundee, Dow Street, Dundee DD1 5EH, United Kingdom; 8 Department of Internal Medicine, University of Tübingen, Otfried-Müller-Straβe 10, 72076 Tübingen, Germany; 9 Department of Pathology and Molecular Medicine, McMaster University, 1280 Main Street West, Hamilton, Ontario L8S4K1, Canada; 10 Centre for Innovation, Canadian Blood Services, 1280 Main Street West, Hamilton, Ontario L8S4K1, Canada; 11 Institute of Agricultural and Nutritional Sciences, Martin Luther University Halle-Wittenberg, Von-Danckelmann-Platz 2, 06120 Halle (Saale), Germany

## Abstract

The mitogen- and stress-activated kinase MSK1/2 plays a decisive role in apoptosis. In analogy to apoptosis of nucleated cells, suicidal erythrocyte death called eryptosis is characterized by cell shrinkage and cell membrane scrambling leading to phosphatidylserine (PS) externalization. Here, we explored whether MSK1/2 participates in the regulation of eryptosis. To this end, erythrocytes were isolated from mice lacking functional MSK1/2 (*msk*^−/−^) and corresponding wild-type mice (*msk*^+/+^). Blood count, hematocrit, hemoglobin concentration and mean erythrocyte volume were similar in both *msk*^−/−^ and *msk*^+/+^ mice, but reticulocyte count was significantly increased in *msk*^−/−^ mice. Cell membrane PS exposure was similar in untreated *msk*^−/−^ and *msk*^+/+^ erythrocytes, but was enhanced by pathophysiological cell stressors *ex vivo* such as hyperosmotic shock or energy depletion to significantly higher levels in *msk*^−/−^ erythrocytes than in *msk*^+/+^ erythrocytes. Cell shrinkage following hyperosmotic shock and energy depletion, as well as hemolysis following decrease of extracellular osmolarity was more pronounced in *msk*^−/−^ erythrocytes. The *in vivo* clearance of autologously-infused CFSE-labeled erythrocytes from circulating blood was faster in *msk*^−/−^ mice. The spleens from *msk*^−/−^ mice contained a significantly greater number of PS-exposing erythrocytes than spleens from *msk*^+/+^ mice. The present observations point to accelerated eryptosis and subsequent clearance of erythrocytes leading to enhanced erythrocyte turnover in MSK1/2-deficient mice.

The closely related mitogen- and stress-activated kinases MSK1 and MSK2 are involved in signal transduction that governs survival and apoptosis of nucleated cells^1^,^2^,^3^,^4^,^5^,^6^,^7^. Stimulators of MSK1 include the Ras-mitogen-activated protein kinase (MAPK)/p38 MAPK signal transduction pathway^1^,^8^,^9^,^10^. MSK1 participates in a wide array of cellular functions, including regulation of immediate-early gene expression^9^,^11^, an effect attributed to its ability to phosphorylate histone H1 and H3 and thus fostering the modification of chromatin structure^3^,^6^,^9^,^12^. Moreover, MSK1 contributes to the regulation of NF-κB activation^2^,^13^, of cAMP-response element^11^,^14^,^15^, of caspase activity^16^ and of Bad phosphorylation^17^. Furthermore, MSK1/2 deficiency enhances the formation of PGE_2_^18^.

Similar to nucleated cells, erythrocytes may undergo suicidal death or eryptosis, which is characterized by cell shrinkage and cell membrane scrambling^19^. Triggers of eryptosis include activation of Ca^2+^-permeable cation channels^20^,^21^,^22^,^23^,^24^,^25^,^26^, which are activated by PGE_2_^27^. The activation of the channels leads to Ca^2+^ entry, activation of Ca^2+^-sensitive K^+^ channels, exit of KCl with osmotically obliged water and, thus, to cell shrinkage^28^. Cytosolic Ca^2+^ further stimulates scrambling of the erythrocyte membrane with exposure of phosphatidylserine at the cell surface^26^,^29^,^30^,^31^,^32^. The Ca^2+^ sensitivity of cell membrane scrambling is increased by ceramide^33^. Phosphatidylserine exposing erythrocytes are rapidly phagocytosed and thus cleared from circulating blood^34^,^35^,^36^,^37^. Accordingly, accelerated eryptosis enhances the turnover of erythrocytes, which may lead to anemia, if the accelerated loss of erythrocytes is not compensated by a similar increase of erythrocyte formation, which is evident from reticulocytosis^19^.

In the present study, we explored whether MSK1/2 influences the survival of erythrocytes in response to pathophysiological cell stressors such as hyperosmotic shock and energy depletion. To this end, the eryptotic phenotype was characterized in mice lacking functional MSK1/2 (*msk*^−/−^) and their corresponding wild type mice (*msk*^+/+^).

## Results

### Absence of overt anemia but increased reticulocytosis in msk^−/−^ mice

The present study addressed the impact of MSK1/2 on eryptosis in mice. To this end, experiments were performed in mice lacking functional MSK1/2 (*msk*^−/−^) and corresponding wild type mice (*msk*^+/+^). As a first approach, a blood count was performed. As shown in Table 1, erythrocyte count (RBC), hemoglobin concentration (HGB), hematocrit (HCT), mean corpuscular volume (MCV), and mean corpuscular hemoglobin concentration (MCHC) were not significantly different between *msk*^−/−^ than in *msk*^+/+^ mice. Mean corpuscular hemoglobin (MCH) was, however, slightly but significantly increased in *msk*^−/−^ as compared to *msk*^+/+^ mice (Table 1). Reticulocyte count was significantly higher in *msk*^−/−^ than in *msk*^+/+^ mice, pointing to enhanced erythrocyte formation in *msk*^−/−^ mice (Table 1).

### Expression of MSK1 and MSK2 in human and murine erythrocytes

**Figure 1:**
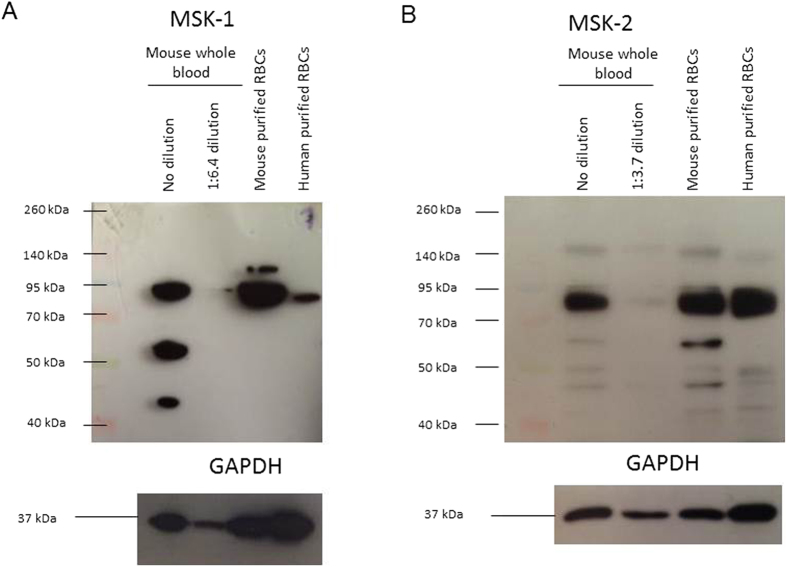
MSK1 and MSK2 expression in murine and human erythrocytes. (**A**) Original Western blots of MSK1 (~90 kDa) and GAPDH (~37 kDa) in murine whole blood (lane 1), 1:6.4 diluted whole blood (lane 2) and purified erythrocyte (RBC) preparation (lane 3) and human erythrocytes (lane 4). (**B**) Original Western blots of MSK2 (~86 kDa) and GAPDH (~37 kDa) in murine whole blood (lane 1), 1:3.7 diluted whole blood (lane 2) and purified erythrocyte (RBC) preparation (lane 3) and human erythrocytes (lane 4).

Immunoblotting was employed to test whether MSK1 and/or MSK2 are expressed in erythrocytes. To this end, erythrocytes from humans or from mice were isolated and purified. Equal amounts of protein lysates were made and immunoblotting was performed. GAPDH served as a loading control. Expression of MSK1 and MSK2 was determined in lysates from murine whole blood and from purified murine erythrocytes. As illustrated in Fig. 1, the incubation with MSK1 and MSK2 specific antibodies both yielded a band of 90 (MSK1) and 86 (MSK2) kDa in murine and human erythrocytes, respectively.

### Increased susceptibility of msk^−/−^ erythrocytes to osmosensitive eryptosis and hemolysis

**Figure 2:**
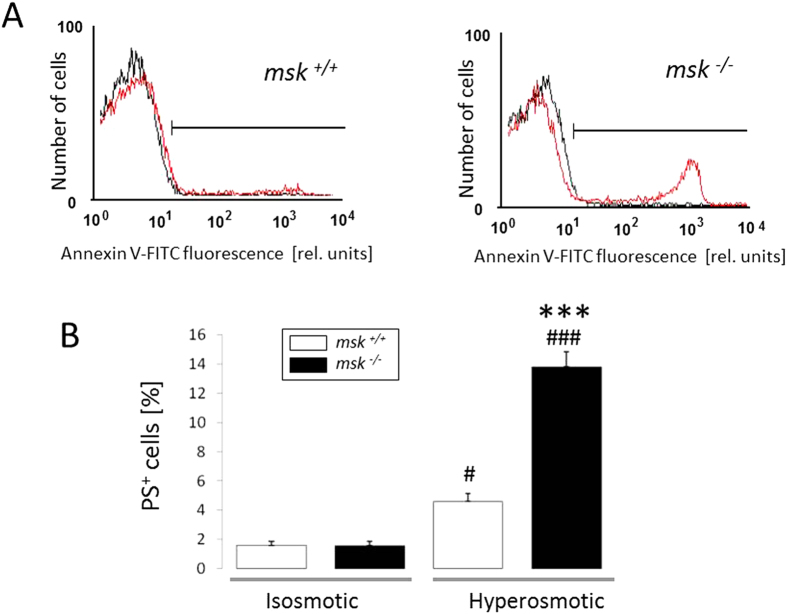
Effect of hyperosmolarity on phosphatidylserine abundance at the surface of erythrocytes from *msk*^−/−^ and *msk*^+/+^ mice. (**A**) Histogram overlay and (**B**) Means ± SEM (n = 7) of annexin V-binding erythrocytes in isosmotic (*black line*) or hyperosmotic (*red line*, +550 mM sucrose) Ringer. ^#,###^(p < 0.05; p < 0.001) from isosmotic, ^***^(p < 0.001) from *msk*^+/+^.

**Figure 3:**
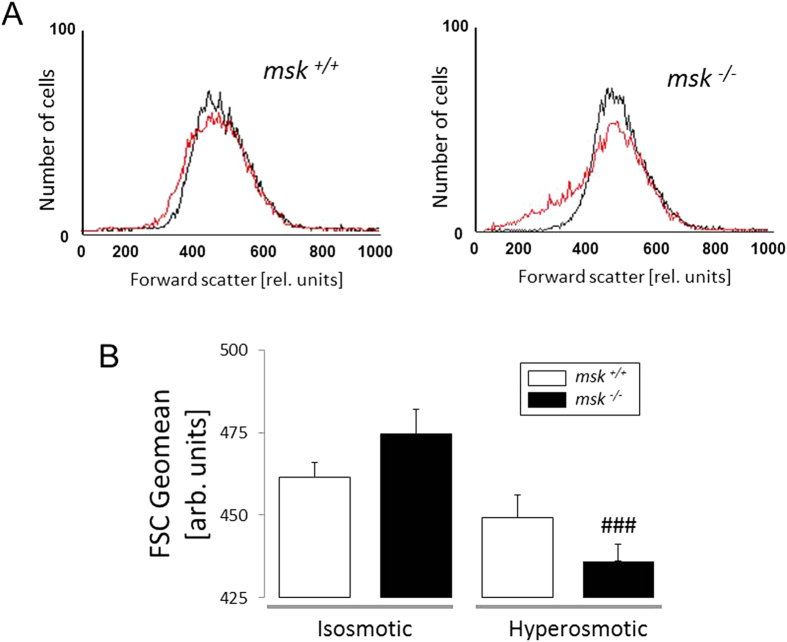
Effect of hyperosmolarity on forward scatter of erythrocytes from *msk*^−/−^ and msk^+/+^ mice. (**A**) Histogram overlay and (**B**) Means ± SEM (n = 7) of erythrocyte FSC Geomean in isosmotic (*black line*) or hyperosmotic (*red line*, +550 mM sucrose) Ringer. ^###^(p < 0.001) from isosmotic.

**Figure 4:**
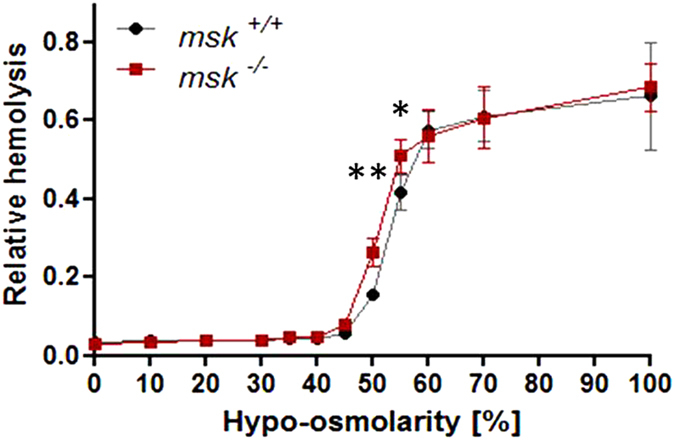
Osmotic resistance of erythrocytes from *msk*^−/−^ and *msk*^+/+^ mice. Means ± SEM (n = 3−4) of relative hemolysis as a function of extracellular osmolarity (% hyposmolar of isomotic Ringer). ^*,**^(p < 0.05, p < 0.01) from *msk*^+/+^.

Further experiments then addressed the susceptibility of MSK1/2-deficient erythrocytes to osmotic shock, a known stimulator of eryptosis, i.e. increase of phosphatidylserine exposure and decrease of cell volume^26^. Prior to osmotic shock, annexin V-binding reflecting phosphatidylserine exposure at the erythrocyte surface was similar in both *msk*^−/−^ and msk^+/+^ erythrocytes (Fig. 2). Following exposure of erythrocytes for 1 h to hyperosmotic Ringer (addition of 550 mM sucrose), however, the annexin V-binding was significantly higher in *msk*^−/−^ than in *msk*^+/+^ erythrocytes (Fig. 2). To depict cell shrinkage, forward scatter of *msk*^−/−^ and *msk*^+/+^ erythrocytes was determined in flow cytometer analysis. As shown in Fig. 3, forward scatter was significantly reduced by hyperosmotic shock in erythrocytes from both *msk*^−/−^ and *msk*^+/+^ mice. The effect, however, tended to be more pronounced in *msk*^−/−^ erythrocytes than in *msk*^+/+^ erythrocytes. Further experiments explored the resistance of erythrocytes to a decline of extracellular osmolarity. As illustrated in Fig. 4, the resistance of erythrocytes to decreases of osmolarity was significantly lower in *msk*^−/−^ than in *msk*^+/+^ mice. Thus, MSK1/2 deficiency enhances the sensitivity of erythrocytes to both hyper- and hypoosmotic shock.

### Increased vulnerability of msk^−/−^ erythrocytes to energy-sensitive eryptosis

**Figure 5:**
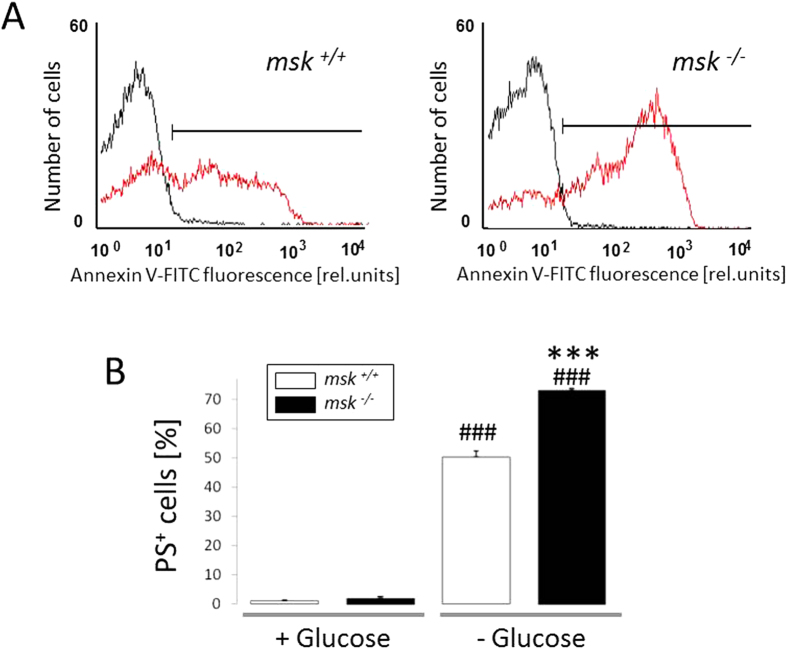
Effect of energy depletion on phosphatidylserine abundance at the surface of erythrocytes from *msk*^−/−^ and *msk*^+/+^ mice. (**A**) Histogram overlay and (**B**) Means ± SEM (n = 3−4) of annexin V-binding erythrocytes in glucose-containing (*black line*, +Glucose) or glucose-depleted (*red line*, −Glucose) Ringer. ^###^(p < 0.001) from +Glucose. ^***^(p < 0.001) from *msk*^+/+^.

**Figure 6:**
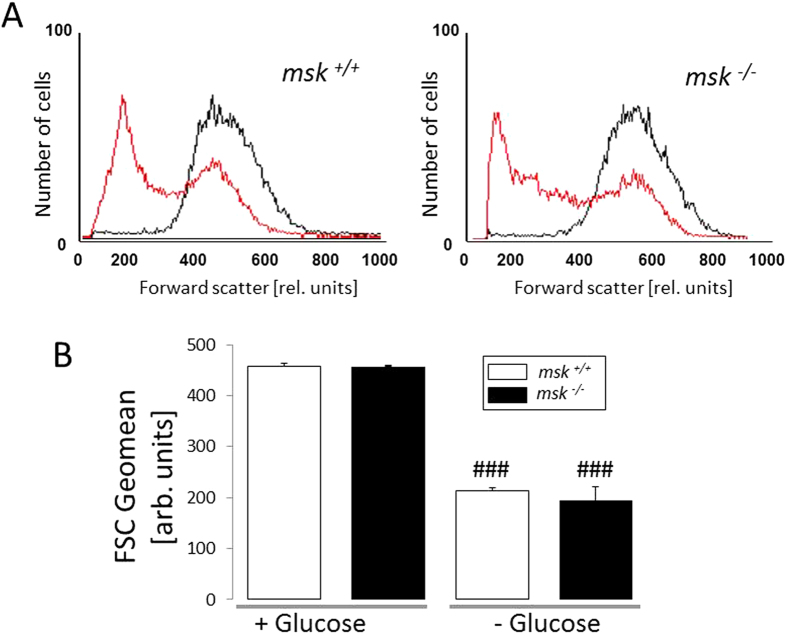
Effect of energy depletion on forward scatter in erythrocytes from *msk*^−/−^ and *msk*^+/+^ mice. (**A**) Histogram overlay and (**B**) Means ± SEM (n = 3−4) of erythrocyte FSC Geomean from glucose-containing (*black line*, +Glucose) or glucose-depleted (*red line*, −Glucose) Ringer. ^###^(p < 0.001) from +Glucose.

Additional experiments were performed in the presence and absence of glucose, as energy depletion is known to foster eryptosis^38^. As shown in Fig. 5, annexin V-binding reflecting phosphatidylserine exposure at the erythrocyte surface was significantly increased by 12 h glucose depletion, an effect significantly higher in *msk*^−/−^ than in *msk*^+/+^ erythrocytes. Furthermore, as shown in Fig. 6, forward scatter was significantly reduced by energy depletion in erythrocytes from both *msk*^−/−^ and *msk*^+/+^ mice. This effect tended to be larger in *msk*^−/−^ than in *msk*^+/+^ erythrocytes, an effect, however, not reaching statistical significance (Fig. 6).

### Enhanced *in vivo* clearance and entrapment of eryptotic erythrocytes in the spleens of msk^−/−^ mice

**Figure 7:**
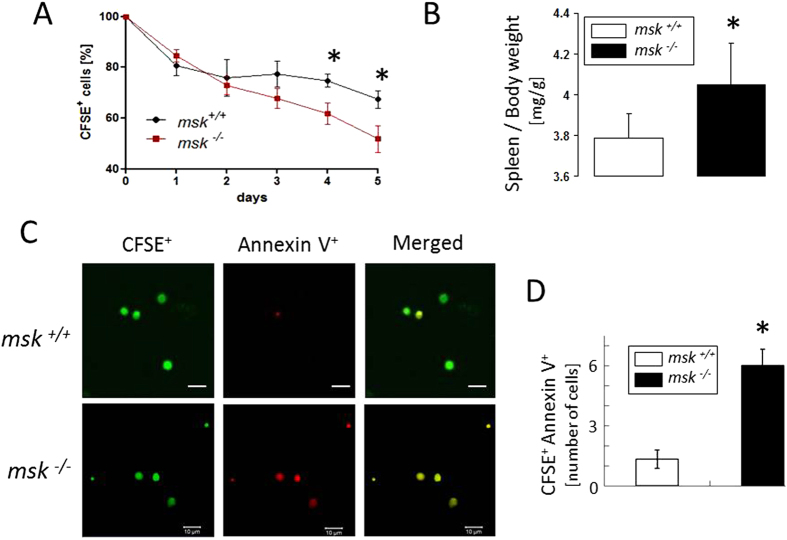
Enhanced clearance and splenic entrapment of eryptotic erythrocytes in *msk*^−/−^ mice. (**A**) Means ± SEM (n = 3−4) of the percentages of autologously-injected circulating CFSE-labeled erythrocytes plotted against time. (**B**) Means ± SEM of the spleen/body weight ratio (mg/gram) of *msk*^−/−^ (n = 21) and *msk*^+/+^ (n = 33) mice. (**C**) Confocal images of CFSE-dependent (*left panels*), annexin V-dependent (*middle panels*) and merged fluorescence (*right panels*) and (**D**) Means ± SEM (n = 3−4) of number of CFSE and annexin V positive splenic erythrocytes from msk^−/−^ and *msk*^+/+^ mice. ^*^(p < 0.05) from *msk*^+/+^.

Eryptotic erythrocytes are rapidly cleared from circulating blood^36^. Thus, additional experiments were performed to disclose a possible effect of MSK1/2 deficiency on erythrocyte clearance. To determine the life span of circulating erythrocytes, blood was drawn from *msk*^−/−^ and *msk*^+/+^ mice and erythrocytes were labelled with CFSE and injected autologously in the mice of the respective genotype. As shown in Fig. 7A, within 4 and 5 days CFSE-labeled *msk*^−/−^ erythrocytes disappeared from circulating blood of *msk*^−/−^ mice more rapidly than CFSE-labeled *msk*^+/+^ erythrocytes from circulating blood of *msk*^+/+^ mice. Thus, the life span of *msk*^−/−^ erythrocytes in *msk*^−/−^ mice was significantly shorter than the life span of *msk*^+/+^ erythrocytes in *msk*^+/+^ mice. The labelled erythrocytes were mainly trapped in the spleen. The ratio of spleen weight to body weight was slightly but significantly larger in *msk*^−/−^ mice as compared to *msk*^+/+^ mice (Fig. 7B). The number of fluorescent annexin V-binding and thus phosphatidylserine-exposing erythrocytes as visualized by fluorescence confocal microscopy was again higher in the spleens from *msk*^−/−^ mice than in the spleens from *msk*^+/+^ mice reflecting enhanced trapping of eryptotic erythrocytes in *msk*^−/−^ mice (Fig. 7C,D).

## Discussion

According to the present observations, a lack of MSK1/2 enhances the susceptibility of erythrocytes to undergo suicidal erythrocyte death or eryptosis following pathophysiological cell stressors such as hyperosmotic shock and energy depletion. The MSK1/2-deficient (*msk*^−/−^) mice did not exhibit overt anemia but showed marked increase in erythrocyte turnover that contributes to a mild increase in splenic mass. Moreover, the erythrocytes from *msk*^−/−^ mice are more sensitive than erythrocytes from *msk*^+/+^ mice to triggers of eryptosis, including hyperosmotic shock and energy depletion. On the other hand, MSK1/2 deficiency decreases the resistance against hemolysis following decrease of extracellular osmolarity. Apparently, MSK1/2 deficiency increases the sensitivity of erythrocytes to both cell shrinkage and cell swelling.

Hyperosmotic shock and energy deletion trigger eryptosis only in a subset of the erythrocyte population, indicating that the circulating erythrocytes are not uniformly sensitive to those triggers of eryptosis. As a matter of fact, the susceptibility of circulating erythrocytes towards triggers of eryptosis increases with erythrocyte age^39^,^40^. On the other hand, evidence has been reported that newly formed erythrocytes are highly susceptible to suicidal death, a phenomenon called neocytolysis^41^,^42^,^43^. Along those lines, considerable diversity of lysophosphatidic acid (LPA) induced Ca^2+^ influx and phospatidylserine translocation was observed in seemingly morphologically homogeneous erythrocyte populations^44^. The Ca^2+^ response to LPA was virtually lacking in reticulocytes and still highly variable in old erythrocytes^44^.

Collectively, the present observations highlight the significance of MSK1/2 for erythrocyte survival. Phosphatidylserine-exposing cells are bound to macrophages^45^, engulfed and degraded^46^, and thus rapidly cleared from circulating blood^36^,^37^. Along those lines, *msk*^−/−^ erythrocytes are cleared more rapidly from the circulation. The accelerated erythrocyte death and clearance from circulating blood is outweighed by compensatory increase of erythropoiesis in *msk*^−/−^ mice, which is reflected by increased numbers of circulating reticulocytes in those mice.

Mechanistically, exposure of erythrocytes to hypertonic extracellular environment *in vitro* simulates the osmotic conditions encountered in the kidney medulla. Under pathological conditions such as acute renal failure, erythrocytes are trapped in the kidney medulla, thus predisposing erythrocytes to eryptosis^33^. It is, therefore, tempting to speculate that MSK1/2 influences erythrocyte survival and its ramifications in systemic conditions such as renal failure. The MSK1/2 upstream molecule p38 MAPK orchestrates adaptation to hypertonicity in mammalian cells^47^,^48^. In nucleated cells, hypertonic shock modulates cAMP response element-binding protein *via* activation of MSK1-dependent signaling^49^. In erythrocytes, a similar parallel can be drawn as hyperosmotic shock elicits phosphorylation of p38 MAPK that regulates the eryptosis machinery^50^. The *msk*^−/−^ erythrocytes have further an enhanced sensitivity to the eryptotic effect of cellular energy deprivation, another powerful stimulator of eryptosis^38^. Signaling involved in the regulation of eryptosis following cellular energy depletion includes protein kinase C, AMP activate kinase and Janus kinase 3^19^.

According to the present data MSK1/2 contributes to both osmo- and energy-sensitive regulation of erythrocyte survival. Without stimulation of eryptosis, the percentage of eryptotic cells is similar in *msk*^−/−^ mice and in *msk*^+/+^ mice. The susceptibility of the erythrocytes from *msk*^−/−^ mice to eryptosis is, however, apparent following osmotic shock and energy depletion. Eryptosis is enhanced by erythrocyte age, a wide variety of anemia-causing xenobiotics and endogenous substances^19^ and several clinical disorders, including iron deficiency, phosphate depletion, hepatic failure, dehydration, fever, Hemolytic Uremic Syndrome, end stage renal disease, sepsis, malaria, malignancy and Wilson’s disease^19^,^51^. Eryptosis may further influence erythrocyte storage for transfusion^52^. MSK1 deficiency may enhance the susceptibility to the eryptotic effect of those xenobiotics, endogenous substances and clinical disorders. In view of the accelerated clearance of erythrocytes and a mild splenomegaly in *msk*^−/−^ mice, triggers of eryptosis are apparently operative in the blood of those mice.

Phosphatidylserine-exposing erythrocytes adhere to the vascular wall^53^,^54^,^55^,^56^,^57^ and to other erythrocytes^58^; they further stimulate blood clotting^53^,^59^,^60^. Thus, excessive eryptosis may compromise microcirculation. Along those lines, enhanced eryptosis has been suggested to participate in the vascular injury of metabolic syndrome^61^.

In conclusion, lack of MSK1/2 leads to enhanced susceptibility to suicidal erythrocyte death or eryptosis following osmotic shock and energy depletion leading to accelerated splenic trapping of circulating erythrocytes.

## Materials and Methods

### Human erythrocytes

Highly purified erythrocyte concentrates were provided by the blood bank of the University of Tübingen. The erythrocyte concentrates were virtually free of white blood cells and contained less than 1% platelets. The Committee approving the experiments, in name, is the ethics committee of the University of Tübingen, given report number: 184/2003V. Informed consent was obtained from all subjects.

### Mice

Experiments were performed in 9- to 16-wk-old MSK1/2-deficient mice (*msk*^−/−^) as well as sex-and age matched wild-type mice (*msk*^+/+^) which were fed a control diet (C1314; Altromin, Heidenau, Germany) and had access to drinking water *ad libitum*. The *msk*^−/−^ mice have been described previously^15^,^18^. The animals were maintained under specific pathogen-free conditions and all experiments described in the methods were carried out in accordance with the approved guidelines (American Physiological Society as well as the German law and the EU Animals Scientific Procedures Act for the welfare of animals) and were approved by local authorities of the state of Baden-Württemberg.

### Blood count and isolation of murine erythrocytes

For all experiments except for the blood count, heparin blood was retrieved from the retrobulbar plexus of mice^62^. For the blood count, EDTA blood was analyzed using an electronic hematology particle counter (type MDM 905 from Medical Diagnostics Marx; Butzbach, Germany) equipped with a photometric unit for haemoglobin determination. To obtain pure erythrocytes, murine erythrocytes were separated utilizing Ficoll (Biochrom AG, Germany) and washed twice with Ringer solution containing (in mM): 125 NaCl, 5 KCl, 1 MgSO_4_, and 32 HEPES/NaOH (pH 7.4), 5 glucose, and 1 CaCl_2_.

### Reticulocyte count

For determination of the reticulocyte count EDTA-whole blood (5 μl) was added to 1 ml Retic-COUNT (Thiazole orange) reagent from Becton Dickinson. Samples were stained for 30 min at room temperature, and flow cytometry was performed according to the manufacturer’s instructions. Forward scatter (FSC), side scatter (SSC), and Thiazole orange-fluorescence intensity (in FL-1) of the blood cells were determined. The number of Retic-COUNT positive reticulocytes was expressed as the percentage of the total gated erythrocyte populations. Gating of erythrocytes was achieved by analysis of FSC vs. SSC dot plots using CellQuest software.

### Determination of the osmotic resistance

For measurement of osmotic resistance 2 μl erythrocyte pellets were exposed in a 96 well plate for 2 min to phosphate-buffered saline (PBS) solutions (in mM: 1.05 KH_2_PO_4_, 2.97 Na_2_HPO_4_, 155.2 NaCl) of decreasing osmolarity as prepared by mixing a PBS solution with a defined volume of distilled water. After centrifugation (500 g for 5 min), the Hb concentration of the supernatants was determined photometrically (at 405 nm).

### Incubations and solutions

For *in vitro* analysis of eryptosis, erythrocytes were isolated by washing two times and subsequent incubation *in vitro* at a hematocrit of 0.4% in Ringer solution at 37 °C for the indicated time periods. Where indicated, glucose was removed or sucrose (550 mM) added to the Ringer solution.

### Phosphatidylserine exposure and forward scatter

After incubation, erythrocytes were washed once in Ringer solution containing 5 mM CaCl_2_. The cells were then stained with annexin V-FITC (1:250 dilution; Immunotools, Friesoythe, Germany) at a 1:500 dilution. After 15 min, samples were measured by flow cytometric analysis (FACS-Calibur; BD). Cells were analyzed by forward scatter, and annexin V-fluorescence intensity was measured with an excitation wavelength of 488 nm and an emission wavelength of 530 nm on a FACS calibur (BD, Heidelberg, Germany).

### Measurement of the *in vivo* clearance of fluorescence-labeled erythrocytes

The *in vivo* clearance of fluorescence-labeled erythrocytes was determined as described previously^63^. Briefly, erythrocytes (obtained from 200 μl blood) were fluorescence-labeled by staining the cells with 5 μM carboxyfluorescein-diacetate-succinimidyl-ester (CFSE) (Molecular Probes, Leiden, Netherlands) in PBS and incubated for 30 min at 37 °C. After washing twice in PBS containing 10% FCS the pellet was resuspended in Ringer solution (37 °C), and 100 μl of the CFSE-labelled erythrocytes (50% hematocrit) were injected into the tail vein of the recipient mouse. As indicated, blood was retrieved from the tail veins of the mice, and CFSE-dependent fluorescence intensity of the erythrocytes was measured as described above. The percentage of CFSE-positive erythrocytes was calculated in % of the total labelled fraction determined 10 min after injection.

### Confocal microscopy

For the detection of annexin V-binding and CFSE-dependent fluorescence of erythrocytes in the spleen, the spleens of *msk*^−/−^ and *msk*^+/+^ mice were homogenized mechanically in 1 ml cold PBS. The suspension was then centrifuged at 500 g for 10 min at 4 °C. The cell pellet was resuspended in 200 μl cold PBS. Five μl of Annexin V-APC (BD, Heidelberg, Germany) were added, and incubation was carried out for 20 min at 37 °C protected from light. Then, the suspension was transferred onto a glass slide and mounted with Prolong® Gold antifade reagent (Invitrogen). Images were taken on a Zeiss LSM 5 EXCITER Confocal Laser Scanning Microscope (Carl Zeiss MicroImaging GmbH, Germany) with a water immersion Plan-Neofluar 63/1.3 NA DIC.

### Immunoblotting

To remove the haemoglobin, 200 μl erythrocyte pellet (1 × 10^9^ cells) were haemolysed in 50 ml of 20 mM HEPES/NaOH (pH 7.4) containing 1 complete protease inhibitor cocktail (Roche). Ghost membranes were pelleted (20,000 g for 20 min at 4 °C) and lysed in 200 μl lysis buffer (125 mM NaCl, 25 mM HEPES/NaOH (pH 7.4), 10 mM Na_2_-EDTA, 10 mM NaF, 10 mM Na-pyrophosphate tetrabasic decahydrate, 0.1% sodium dodecyl sulfate (SDS), 0.5% deoxycholic acid, 1% Triton X-100, 0.4% β-mercaptoethanol and 1 complete protease inhibitor cocktail. Lysed ghost membranes were solubilized in Laemmli sample buffer at 95 °C for 5 min and stored at −20 °C. The murine erythrocytes were washed after isolation from full blood by a single purification step with Ficoll and then lysed in the same lysis buffer as above.

For each lane, equal amounts of protein were loaded and resolved by 8–10% SDS-PAGE precast gel (Invitrogen). For immunoblotting, proteins were electrotransferred onto a PVDF membrane and blocked with 5% non-fat milk in TBS-0.1% Tween 20 (TBS-T) at room temperature for 1 h. The membrane was incubated with rabbit anti-MSK1 (C27B2; #3489) antibody (1:500; 90 kDa) (Cell signaling, USA) or rabbit anti-MSK2 (NBP2-30079) antibody (1:1000; 86 kDa, Novus Biological, USA) or 1:1000 anti-GAPDH antibody (1:1000; 37 kDa, Cell Signaling) at 4 °C overnight in 5% BSA. After washing with TBS-T the blots were incubated with secondary anti-rabbit antibody (1:2000; Cell Signaling) for 1 h at room temperature. After washing, antibody binding was detected with the ECL detection reagent (Life technologies, Germany).

### Statistics

Data are expressed as arithmetic means ± SEM, and statistical analysis was made using ANOVA or t-test, as appropriate. n denotes the number of different erythrocyte specimens studied.

## Additional Information

**How to cite this article**: Lang, E. *et al.* Accelerated apoptotic death and *in vivo* turnover of erythrocytes in mice lacking functional mitogen- and stress-activated kinase MSK1/2. *Sci. Rep.* **5**, 17316; doi: 10.1038/srep17316 (2015).

## Figures and Tables

**Table 1:** Blood count and reticulocyte number in *msk* ^−/−^ and *msk* ^+/+^ mice.

Parameter	*msk*^+/+^	*msk*^−/−^	Units
RBC	12.0 ± 0.5	11.4 ± 0.6	×10^6^/μl
HGB	15.9 ± 0.6	15.7 ± 0.9	g/dl
HCT	45.8 ± 4.9	43.5 ± 5.4	%
MCV	37.8 ± 2.7	37.3 ± 3.1	fl
MCH	13.3 ± 0.1	13.8 ± 0.1*	pg
MCHC	36.3 ± 2.6	38.5 ± 3.1	g/dl
RTC	3.5 ± 0.3	4.7 ± 0.3*	%

Means ± SEM (n = 3−7) of erythrocyte count (RBC), haemoglobin concentration (HGB), haematocrit (HCT), mean corpuscular volume (MCV), mean corpuscular haemoglobin (MCH), mean corpuscular hemoglobin concentration (MCHC), reticulocyte count (RTC) of 9–16 week-old MSK1-deficient mice (*msk*^−/−^) and wild type mice (*msk*^+/+^). * significant (p < 0.05) differences between genotypes (*t*-test).
